# Case of Fungus Hæmatodes of the Kidnies

**Published:** 1814-07

**Authors:** Thomas F. Rance

**Affiliations:** 4, City Road, Finsbury-square


*For the Medical and Physical Journal.*

### *Case of Fungus Hæmatodes of the Kidnies; by* Mr, Thomas F. Rance.

HOWEVER true it may be that some diseases baffle the skill of the medical practitioner, resisting all his curative and palliative attempts, yet these disappointments ought never so to influence his mind as to damp his ex-

 ertions, ertions, or restrain his endeavours in acquiring a more accurate knowledge of the malady under which his patient labours; for as diseases become the more difficult to remove, so should his researches become the more unwearied and assiduous; and if, from the nature of the complaint, his attempts at restoration or relief prove unavailing, he will generally find some recompense from the knowledge he has acquired in forming a more decisive diagnosis and correct prognosis.

The disease to which I am about to engage your attention, has not till of late years attracted the notice of the profession; and I believe we are indebted to that indefatigable practitioner Mr. Hey, of Leeds, whose investigations have tended much to the advancement of the surgical department of the profession: he (as may be seen in his practical work on surgery) first discriminated the fungus hæmatodes from true cancer, and from its fungated appearance and frequent hæmorrhages named it fungus hæmatodes. With respect to the success which attended his treatment, he candidly informs us that his attempts in this respect generally proved abortive; that neither the internal exhibition nor external application of any remedy subdued the disease, nor did the excision of the morbid part; and in a very few instances did the amputation of a limb when affected, prevent the return of the disease so as to save the life of the patient.

Having lately met with a case of fungus hæmatodes, which considering the age of the patient had acquired a large bulk, I feel a desire to communicate the particulars to my brethren of the profession, through the medium of your widely-circulated Journal; not because I am able to recommend any thing new as it respects the treatment of this malady, but as I had an opportunity of examining the subject after death, I am enabled to state the appearance on dissection, which may prove interesting to the anatomist; and as some of your readers may be desirous of being acquainted with the symptoms and treatment while living, I shall proceed to state them as accurately as I am able.

The subject of this paper was a female child of Capt. B.'s, residing near the Commercial Road, aged one year and five months. She was delicate and slender from her birth; her complexion was fair and pale, and her hair light coloured, her habit of body generally costive, and her respiration short and quick. Mrs. B. having, about the middle of January 1813, discovered a lump in her child's side, became alarmed, and requested me to examine it, informing me that she could not account for its production in any other way than by a fall from a table to the floor. On examination, I found found a swelling situated in the left hypochondrium inclined toward the spine, which, although scarcely perceptible to the eye, yet when touched by the finger might be distinctly felt about the size of a hen's egg, which had no pain except it were forcibly pressed. As the child had always been thin, and of a very spare habit, its debility did not at first attract the attention of its friends.

The tumour being situated near the spleen, I thought it probably might be a chronic enlargement of that organ, in consequence of inflammation; on which account, on the 16th January, I ordered blood to be taken from the neighbourhood of the affected part, by means of four leeches. As the skin was hot, the tongue dry, and the bowels constipated, I ordered the following alterative:

℞. Pulvis Scam. Comp. gr. x.
Hydrarg. Submur. gr. iv. tere simul bene et divide in chartulas quatuor æquales.
Capt. unam alterâ quâque mane.

Jan. 31.—As the bowels were inactive, I substituted the following:

℞. Crem. Tart. in Pulv.
Pulv. Rad. Jalap, āā. gr. xv. div. in chart. tres.
Capt. unam tertiâ quâque mane.

Feb. 5.—The febrile symptoms were considerably increased, the pulse was quick, the belly costive, and the tongue very much parched (during the whole of the time the child laboured under the disease, the tongue was quite clean, and of a bright red colour).

℞. Hydrarg. Submur. gr. ij.
Pulv. Rad. Jalap. gr. viij. div. in ch. iv.
Capt. unam omni mane.

13th.—The swelling appeared to have increased a little; pulse again quick; mouth very dry; urine natural in quantity and appearance. (The urine throughout the whole of the disease was natural in quantity: toward the latter stage of the complaint it became paler in colour than usual, and was twice bloody: the quantity of urine has been a matter of surprise to me, as the organic structure of both kidnies seemed to be nearly destroyed.)

℞. Pulv. Scammon. Comp. gr. v.
Hyd. Submur. gr. j. ft.
Pulvis mane sumend.

℞. Succi Limon. Recentis, ℥iij.

Kali ppt. gr. xv.

Aq. ℥viij.

Syrup. Croci, ℥i. M. ft.

Mist. Cap. Coch. i. med. 4tis horis.

Feb.

Feb. 15th.—As the tumour increased very much in size, I requested Mrs. B. to take the advice of Dr. Babington, who prescribed the following medicines:

℞. Hyd. Submur. gr. iij.

Sodæ Subcarbon, ℈ij.

Pulv. Cretæ Comp. ʒi. div. in chart. xij.

Capt. i. nocte maneque.

℞. Linim. Hydrarg. (Ph. novæ) ℈j.

Fricetur pars affecta omni nocte.

19th.—Dr. B. again saw her, and prescribed the following mixture:

℞. Lactis Amygd. ℥ifs.
Sodæ Subcarb. ℈i. M.
Capt. Coch. i. med. bis. indie.
Pergat in usu Linimenti Hydrarg. et Applicetur
Emplastrum Hydrarg. parti affectæ.

20th.—Having a troublesome cough, I gave her a little of the Mucilago Acaciæ cum Syrup. papav. which had the desired effect.

April 2d.—As the cough was removed, I desired Mrs. B. to persevere in the last prescription of Dr. Babington.

On the 12th of June the febrile symptoms again returned, owing to a constipated state of the bowels. I gave an aperient powder, which relieved the symptoms.

15th.—The plaster came off in consequence of its having lost its adhesive quality. As the child found relief from its supporting the weight of the tumour, I ordered it to be renewed. She now preferred laying on her right side.

July 3d.—As the child began to loathe the medicines, and shrink from the friction of the liniment, I requested them to be discontinued.

About the 9th of July a tumour began to make its appearance in the right hypochondrium, seemingly from the lower edge of the liver: its situation was so contiguous to that viscus, that it was almost impossible to distinguish it from it.

As I had seen some benefit derived from the use of Calomel and Cicuta in the glandular enlargements of strumous habits, I deemed it adviseable to try their combined effects in this case, and about the 10th ordered them in the following form:

℞. Hyd. Submur. gr. iv.

Pulvis Conii, gr. xij.

Sacch. alb. ʒfs. tere simul et div. in chart. vi. æquales.

Capt. unam nocte maneque.

These, with the application of the Emplast. Hydrarg. I continued till the 7th of August. The urine now became limpid, but natural in quantity.

9th.

9th.—The child voided a considerable quantity of bloody urine, which I concluded arose from the rupture of a blood-vessel in one of the kidnies. Feverish symptoms were again present, with constipation of the bowels.

℞. Infus. Rosæ, ℥ifs.

Magnesiæ Sulph. ʒifs.

Syr. Simpl. ʒifs. M.

Capt. Coch. i. med ter in die.

13th.—The hæmaturia was nearly abated; the urine was now but little tinged with blood. As the bowels were but slightly acted upon, I added ʒfs Magnesiæ Sulphatis to the mixture.

17th.—Urine more natural, and bowels less constipated. Rep. Infus. Rosæ, &c. As this mixture had the desired effect in procuring alvine evacuations, and abating febrile action, and as there seemed no possibility of reducing the diseased enlargement, I only desired this to be given occasionally.

Nov. 4th.—An irritable state of the stomach came on, that organ regurgitating its contents as soon as food was swallowed, to allay which I gave the following mixture:

℞. Conf. Arom. gr. x.
Aq. Cinnamom. ℥i.
Syr. pap. alb. ʒi. M.
Capt. Coch. i. med 4tis horis.

Nov. 10th.—The child was seized with violent fits of pain, often shrieking out, and was continually picking its nose and ears.

℞. Tinctur. Opii, gtt. xij.

Aq. Puræ, ℥i.

Syr. pap. alb. ℥fs. M.

Capt. Coch. i. min ter in die vel sæpius si dolor urgeat.

22d.—The hæmaturia having returned, accompanied with the same symptoms as before, I again had recourse to the Infus. Rosæ, &c. which had the desired effect.

Dec. 8th.—The tumours on each side now became evidently much larger; that on the left side was now so tender, that the pressure of the plaster gave pain; I therefore ordered its removal. So sensible was the part to pain that the child screamed violently when it was touched by the finger.

Jan. 10th, 1814.—The swellings were still increased; that on the left side now formed a very considerable projection between the umbilicus and the anterior superior spinous process of the ilium. The projection felt as if it were the apex of the tumour.

23d.—The bowels were again constipated. As it was necessary to make the medicine as palatable as possible, owing to to the difficulty there was in getting her to swallow it, I gave the following:

℞. Infus. Sennæ, ℥ifs.

Fructus Tamarind, ʒij.

Man. Opt. ʒi.

Crem. Tart. ʒfs. M.

Capt. Coch. i. med. mane et rep. p. r. n.

Feb. 14th.—The child now took very little food, and would not swallow any thing except it were highly seasoned. She craved continually for ardent spirits, which a servant had imprudently been in the habit of giving her.

16th.—The pain and tenderness of the affected parts were now very great; and the symptoms of irritation were so high as to cause her to pick her nose and ears till they became quite raw and bled.

21st.—The pulse became much quicker and smaller. Both the upper and lower extremities were œdematous. There were now evident signs of approaching dissolution, and the 25th finally closed the scene of her sufferings.

*Appearances on Dissection.*—Having divided and turned aside the loose parietes of the abdomen, a large tumour presented itself, which seemed to occupy a considerable part of the cavity of the abdomen, having the appearance of the heart when the pericardium is divided. It extended from the diaphragm, which was thrust upward into the thorax, down to the cavity of the pelvis on the left side. None of the intestines were before it, except the great arch of the colon, which strongly adhered to its upper surface. On the left side a tumour was seen rising from under the former, and was in a great measure covered by it. The liver appeared with its lower edge considerably raised upward. The gall-bladder was visible, and completely distended with bile. In endeavouring to remove the larger tumour, I found that it strongly adhered to the lesser, as well as to the mesentery, mesocolon, glandulæ renales, &c. &c. Having accomplished its removal, I proceeded to examine the tumour on the right side, which strongly adhered to the cæcum, the appendix vermiformis being quite imbedded in it. I made a longitudinal incision into it, when a substance of a medullary appearance and consistence escaped. The tumour was so soft, I found it impossible to persevere in any dissection of it. The other viscera of the abdomen and pelvis were in a healthy state, but most of them were thrust from their usual situation. The tumour of the left side, which weighed two pounds twelve ounces avoirdupoise, I took an opportunity of showing to Mr. Astley Cooper, who kindly injected it,

A longitudinal incision being made into it, no appearance of its former renal structure could be observed; but it clearly exhibited three distinct stages of the fungated disease. The posterior surface, although much increased in size, retained its original shape and livid colour. The anterior surface bore but little resemblance to a kidney. The emulgent vessels were considerably increased in size, and the ureter was much distended.

Having recited the particulars of this case, according to the best of my knowledge, I now submit them to your consideration, and, if judged worthy of insertion in your Journal, I shall esteem it as conferring an addition to all former favours. THOMAS F. RANCE.

4, *City Road, Finsbury-square,*

*May* 11, 1814.

